# High sensitivity of summer temperatures to stratospheric sulfur loading from volcanoes in the Northern Hemisphere

**DOI:** 10.1073/pnas.2221810120

**Published:** 2023-11-06

**Authors:** Andrea Burke, Helen M. Innes, Laura Crick, Kevin J. Anchukaitis, Michael P. Byrne, William Hutchison, Joseph R. McConnell, Kathryn A. Moore, James W. B. Rae, Michael Sigl, Rob Wilson

**Affiliations:** ^a^School of Earth and Environmental Sciences, University of St Andrews, St Andrews KY16 9TS, United Kingdom; ^b^School of Geography, Development and Environment and Laboratory of Tree-Ring Research, University of Arizona, Tucson, AZ 85721; ^c^Division of Hydrologic Sciences, Desert Research Institute, Reno, NV 89512; ^d^Department of Atmospheric Science, Colorado State University, Fort Collins, CO 80523; ^e^Climate and Environmental Physics & Oeschger Centre for Climate Change Research, Universtity of Bern, Bern 3012, Switzerland

**Keywords:** volcanoes, climate, ice cores, sulfur isotopes

## Abstract

The 540s, 1450s, and 1600s represent three of the five coldest decades in the Common Era (CE). In each of these cases, the cause of these cold pulses has been attributed to large volcanic eruptions. However, the provenance of the eruption and magnitude of the volcanic forcing remains uncertain. Here, we use high-resolution sulfur isotopes in Greenland and Antarctic ice cores measured across these events to provide a means of improving sulfur loading estimates for these eruptions. In each case, the largest reconstructed tree-ring cooling is associated with an extratropical eruption, and the high-altitude stratospheric sulfate loading of these events is substantially smaller than previous estimates (by up to a factor of two). These results suggest an increased sensitivity of the reconstructed Northern Hemisphere summer temperature response to extratropical eruptions. This highlights the importance of climate feedbacks and processes that amplify and prolong the cooling signal from high latitudes, such as changes in sea ice extent and ocean heat content.

## Significance

Stratospheric sulfate aerosols from large volcanic eruptions reflect incoming sunlight and cool climate, but this process is not well quantified due to a limited observational record. Here, we take advantage of the unique sulfur isotope fingerprint imparted by UV radiation in the upper stratosphere, measuring volcanic sulfate peaks in polar ice cores to determine their climatically important stratospheric component. We find evidence that several of the coldest decades in the last two thousand years—linked with major climatic and societal disruption—were driven by a relatively small amount of stratospheric sulfate from high-latitude eruptions. This challenges the view that tropical eruptions have the largest climatic impacts and suggests enhanced climatic sensitivity to high-latitude volcanic forcing in the Northern Hemisphere.

Volcanic eruptions impart a large negative radiative forcing because their emissions of sulfur dioxide are oxidized into sulfate aerosols which reflect incoming solar radiation and cool the planet (1). The response of the climate system to volcanic eruptions therefore provides a unique opportunity to study the sensitivity of Earth’s climate and associated dynamical feedbacks to repeated short-term external forcing. Only two major eruptions [El Chichón in 1982 CE (Common Era) and Pinatubo in 1991 CE] have occurred during the satellite era, so in order to robustly investigate the climatic response to volcanic forcing over multiple events, it is necessary to use environmental and geochemical archives that record past eruptions.

The most complete record of major volcanic eruptions comes from polar ice cores (e.g., refs. 2–4). Eruptions are marked by pronounced peaks in ice core sulfate (or sulfur) concentration above background levels, representing deposition of volcanic sulfate aerosols transported to the polar regions. When there is a peak in sulfate concentration within age uncertainty in both Greenland and Antarctic ice cores, it is considered a “bipolar eruption” and is commonly assumed to be from a tropical eruption whose plume reached the stratosphere, thus allowing for a global distribution of aerosols (3, 4). Tropical stratospheric eruptions are thought to be more climatically important than higher latitude events because of the global distribution and long residence time (on the order of years) of the sulfate aerosols, which results in an enhanced ability to reflect incoming solar radiation and cool the planet (5). This is in contrast to volcanic eruptions whose plumes remain largely in the troposphere, where aerosols are precipitated out of the atmosphere on the order of weeks, or powerful extratropical eruptions, whose aerosols are generally thought to remain in the hemisphere of the eruption, though direct observations of the large, high-latitude eruptions (e.g., Katmai/Novarupta, Alaska 1912) are scarce (6).

Several major questions remain on the nature of volcanic forcing and climatic response. For instance, a recent comparison of volcanic stratospheric sulfate loading from ice cores with tree-ring estimates of Northern Hemisphere (NH) summer temperatures showed that for the same amount of sulfur injection, the temperature response was larger for extratropical than tropical eruptions (7). Large-scale NH tree-ring reconstructions consistently agree that 536, 1453, and 1601 CE were the coldest summers of the CE (8–11), but these years do not follow the largest sulfur injections which were from the eruptions of Samalas (1257 CE), Tambora (1815 CE) and eruptions in 540 and 1458 CE (4, 12). In particular, the muted climate response in existing climate reconstructions to the 1257 CE eruption of Samalas, the largest known CE eruption, remains a matter of continued debate centered on self-limiting effects from aerosol microphysics, the coemission of ozone-depleting halogens, and limitations of the tree-ring record (10, 13–17). These findings challenge our understanding of the sensitivity of the climate system to sulfate aerosol forcing. Furthermore, the stratospheric sulfate loading that is estimated from ice core sulfate concentrations assumes that all of the sulfate deposited on the ice sheet came via the stratosphere. While this is a valid assumption for large tropical eruptions (due to the distance that needs to be traveled to be deposited near the poles), it has the potential to significantly overestimate the stratospheric sulfate loading for extratropical eruptions that are more proximal to the ice sheet, as was demonstrated for the Katmai 1912 CE eruption using pyrheliometry (6).

Sulfur isotopes in ice cores can be used to identify stratospheric sulfate (18) and thus improve estimates of volcanic forcing of climate. Ultraviolet photochemistry that occurs in the stratosphere at altitudes at or above the ozone layer [in the so-called stratospheric “overworld” above the 380 K isentrope (19)] imparts a mass-independent fractionation (MIF) on volcanic sulfur (18), and this anomalous signature (recorded as a nonzero value of Δ^33^S, see *Materials and Methods*) is preserved and recorded in polar ice cores (18, 20–23). As a result, sulfur isotopes in ice cores provide a means of calculating the proportion of sulfate deposited on the ice sheet that came via the stratospheric overworld (23). Although at high latitudes, the ozone layer is several kilometers above the tropopause, leaving a region called the lowermost stratosphere (LMS) below the ozone layer where sulfur MIF would not be generated (24), modeling results suggest that aerosols from the LMS have a much shorter residence time compared to those in the upper stratospheric overworld (7). Thus, sulfur MIF in ice distinguishes between long residence time sulfate aerosols from the stratospheric overworld and shorter residence time aerosols from the LMS and troposphere and can be used to improve our reconstructions of volcanic forcing to better understand the impacts of these natural “injection” experiments on our climate system.

Here, we use sulfur isotopes to investigate the role of volcanic forcing in three of the coldest decades in the last 2000 y: the 536 and 540 CE eruptions, the 1453 and 1458 CE eruptions, and the eruption around 1600 CE commonly attributed to Huaynaputina volcano in Peru (25, 26). In each case, we use high-resolution sulfur isotopes measured in polar ice cores (Tunu2013 in Greenland and B40 in Antarctica; see ref. 4 for details) across the volcanic event to assess the stratospheric nature of the sulfate aerosols deposited on the ice sheet and to revise the forcings associated with any extratropical events that also deposited sulfate from lower altitudes, where aerosol residence times are short.

## Results and Discussion

### Eruptions from the Late 530s CE.

The major eruptions that occurred in 536 and 540 CE have been linked with a large cooling in the NH and have been associated with societal crises including crop failures and resulting famines, as well as the Justinian plague (4, 27–30). These events serve as an example of the profound impact of volcanism on climate and society and are key targets to improve estimates of volcanic forcing. There are two prominent sulfate peaks at 536 and 540 CE in Greenland ice core records, whereas in most Antarctic records, there is only one prominent sulfate peak around this time [*SI Appendix*, Fig. S1*F*; (31)], which has been shown to be stratospheric (22). Low temporal resolution and postdepositional mixing (i.e., through wind drift) typical at low snow accumulation ice core sites in East Antarctica result in a peak that is so broad that it covers the years 537 to 544 CE, making it difficult to resolve these two sequential eruptions in Antarctica (30, 31). Even at high accumulation sites, cumulative uncertainties in annual-layer dating of ice cores make it challenging to determine confidently which of these eruptions is the bipolar (assumed tropical) eruption, with implications for the size of the sulfur loading and climate forcing associated with these events.

**Fig. 1.**
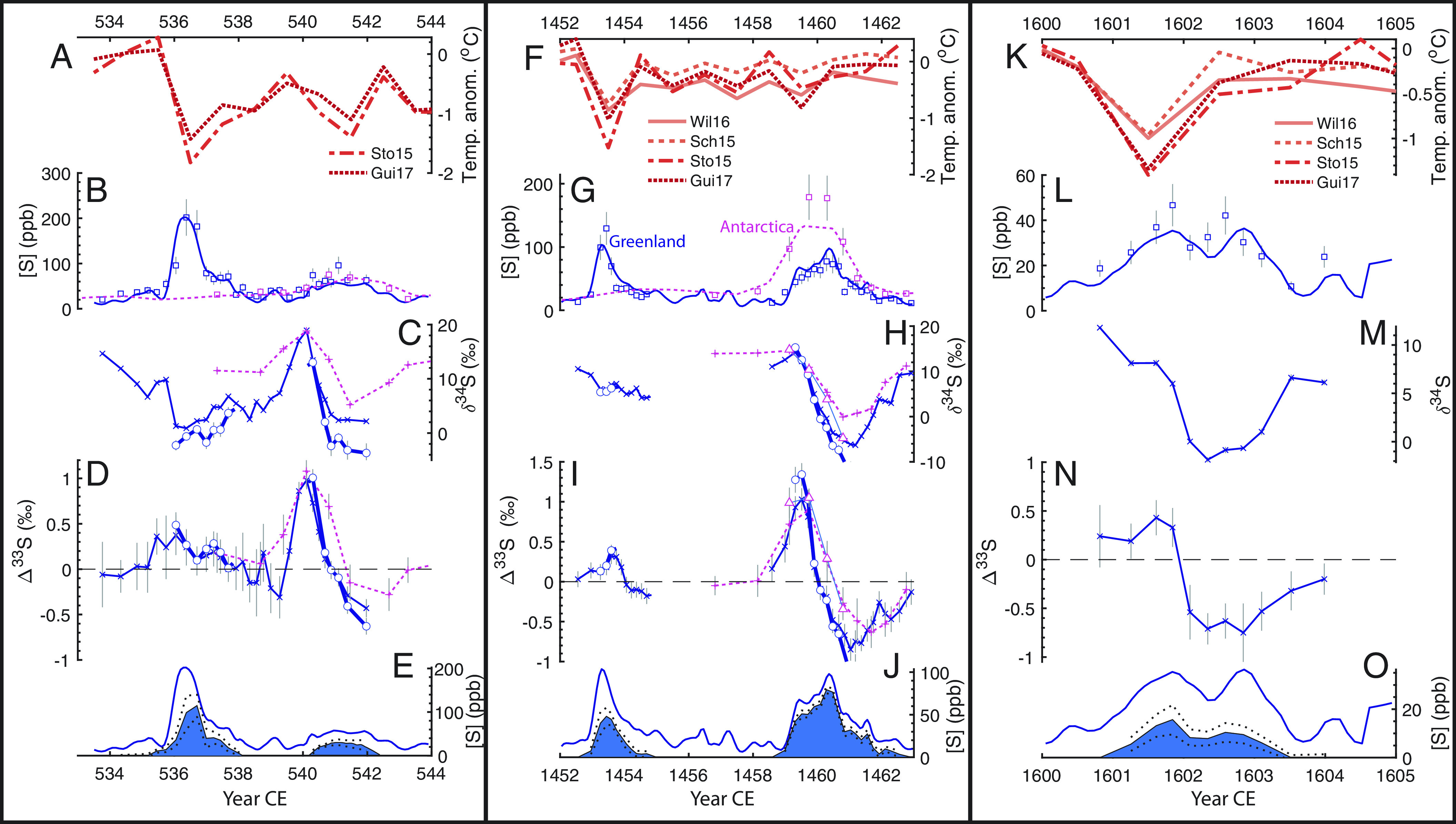
Volcanic events of the 530s (*Left*, *A*–*E*), mid-1450s (*Middle*, *F*–*J*), and 1600s (*Right*, *K*–*O*). (*A*, *F*, and *K*) NH summer temperature anomaly relative to the three-year mean prior to the eruption reconstructed from tree-rings [Wil16 (11), Sch15 (9), Sto15 (10), and Gui17 (33)]. (*B*, *G*, and *L*) Concentration of sulfur (ppb) in the Tunu2013 ice core from Greenland (blue) and the B40 ice core from Antarctica (pink). The line is from continuous measurement on the ice core (4, 31, 34), and squares are from the discrete concentrations made on the isotope samples. (*C*, *H*, and *M*) δ^34^S of sulfate and (*D*, *I*, and *N*) Δ^33^S of sulfate from ice core samples from Tunu2013(blue) and B40 (pink). Measured values are represented by x’s (Tunu2013) or +’s (B40). For samples with more than 65% volcanic sulfate, estimates of the isotopic composition of the volcanic sulfate from isotope mass balance are also plotted in blue circles (Tunu2013) or pink triangles (B40). (*E*, *J*, and *O*) Continuous S concentration (ppb) from Tunu2013 ice core as in (*B*, *G*, and *L*), with the filled blue area as the estimate of the fraction of the sulfate coming from the stratosphere based on isotope mass balance [see *Materials and Methods*; (23)]. The dashed line represents 1σ uncertainty on the fraction of stratospheric sulfate as estimated from Monte Carlo simulations.

Our sulfur isotope data reveal nonzero Δ^33^S in the sulfate from both events in Greenland ice cores, implying that both eruptions were large enough to reach the stratospheric overworld (Fig. 1*D*). Furthermore, we can use the relationship between δ^34^S and Δ^33^S across these events to distinguish between extratropical and tropical eruptions. The initial sulfate deposited in Greenland for the 536 CE event has Δ^33^S = 0 and low values of δ^34^S, consistent with a source of volcanic sulfur that was oxidized and transported to Greenland at altitudes lower than the ozone layer (Fig. 1 *C* and *D*). These isotope signatures require that the volcanic source is proximal to or upwind of the ice sheet and rules out a tropical eruption for the 536 CE event. This distinctive pattern of δ^34^S and Δ^33^S in the initial sulfate deposited on the ice sheet is also seen in the ice core sulfur isotope records for the extratropical eruption of Katmai/Novarupta in Alaska in 1912 CE [(23); *SI Appendix*, Fig. S2], providing additional support for this interpretation. In contrast, when the volcanic sulfate deposited on the ice sheet has been solely transported via the stratospheric overworld, its δ^34^S and Δ^33^S values are highly correlated (r^2^ > 0.9), as shown for known tropical eruptions in Antarctica [Pinatubo 1991 CE, Agung 1963 CE, and Samalas 1257 CE; (20, 32)] and Greenland [Tambora 1815 CE and Samalas 1257 CE, (23)]. Our isotope signatures for the event at 540 CE in Greenland have the same δ^34^S-Δ^33^S correlation and slope as these other known tropical eruptions (*SI Appendix*, Fig. S2). Finally, we also measured the S isotopes across the major sulfate peak in Antarctica around this time, which shows excellent agreement (in terms of the temporal evolution and the absolute values) with the S isotopes measured for the 540 CE event in Greenland (Fig. 1*D*). Given that the magnitude of the sulfur MIF can vary between eruptions (e.g., refs. 22, 23, and 32), we interpret this agreement between the isotope records in both hemispheres to be strong evidence that these sulfate peaks were deposited from the same tropical volcanic event, supporting the bipolar attribution of this event and the ice core chronology.

These conclusions agree with previous work that inferred that the 536 CE event in Greenland was an NH extratropical eruption based on geochemical characterization of tephra from a Greenland ice core that matched volcanic provinces in North America (4). However, the presence of three different geochemical populations in the tephra shards from Greenland meant that no conclusive attribution of the 536 CE event has yet been made (4), although an Icelandic source can be ruled out (35). Based on the identification of the 536 CE event as extratropical, the 540 CE event was concluded to be a large tropical eruption leading to widespread sulfate deposition in both hemispheres, although no tephra has been found associated with this event in Greenland or Antarctica and so no candidate volcano can yet be linked to this event with certainty (4, 36, 37). The similarity in the isotope signatures for the 540 CE event in Greenland and Antarctica supports this inference that the sulfate deposited is from the same volcanic event and thus was a tropical eruption.

### Eruptions from the Mid-1450s CE.

The timing of the eruptions in the 1450s CE, often considered as the inception or intensification of the Little Ice Age (38–40), has become a subject of great debate in recent years. Greenland ice cores have major sulfate peaks in 1453 and in 1458 CE, whereas Antarctic ice cores only have one major sulfate peak around this time, and one minor peak, slightly above background, that can be resolved in some high-resolution cores (34, 41) or in stacked ice core composite records (30) (*SI Appendix*, Fig. S1*G*). Tree-ring-based NH summer temperature reconstructions (9–11, 42–44) have identified 1453 CE as one of the coldest summers of the last millennium. It has therefore been assumed that the large Antarctic sulfate peak was associated with this cooling and was originally assigned an age of 1452 CE (25), with the reasoning that the strongest cooling would occur in the summer after the eruption. This age was subsequently used as a fixed age marker to anchor ice core chronologies across Antarctica (e.g., refs. 45 and 46), and it was widely considered the best age of the Kuwae eruption, which was thought to have occurred in Vanuatu in the mid-15th century (see ref. 47 and references therein). However, recent chronologies developed for high snow accumulation sites in Antarctica based on cm-scale analyses of multiple aerosols have suggested that the event leading to this large sulfate deposition in Antarctica was actually in 1458 CE and that the earlier event in Greenland (in 1453 CE) was connected to the minor sulfate event recorded in some Antarctic cores (34, 48). The dating of these new ice cores has been challenged (e.g., ref. 44), because it resulted in a reduction in the estimate of stratospheric sulfate loading associated with the earlier eruption (from 34 Tg S to 10 Tg S because of comparably small sulfate deposition in Antarctica at this time), and an increase in the estimate of stratospheric sulfur loading associated with the 1458 CE event (from 5.5 to 33 Tg S). This new eruption chronology apparently displaced the volcanic forcing record from the strong cooling response observed in the tree-ring records (44), as the cooling in those reconstructions after 1458 CE is only 0.5 ± 0.2 °C compared to 1 ± 0.3 °C cooling following the eruption in 1453 CE (Fig. 1*F*). Adding to the controversy, geochemical analyses from tephra shards found in an Antarctic ice core around 1458 CE have been used to suggest that the source of the volcanic sulfate in Antarctica in 1458 CE was the result of a high-latitude volcano from South America (Reclus; 49). This tephra calls into question the association of the large Antarctic sulfate peak with the Greenland sulfate peak in 1458 CE, although the sample containing the tephra spanned more than a year in accumulation and thus the tephra could have been from an additional minor eruption at this time.

Our sulfur isotope records provide critical constraints on the timing and nature of these climatically important volcanic events. The presence of nonzero Δ^33^S in both sulfate peaks (Fig. 1*I*) implies that both of the volcanic eruptions that led to the sulfate deposition on the Greenland ice sheet had plumes that reached the stratospheric overworld (18). This finding is consistent with previous work on these two events from Greenland ice cores which also found nonzero Δ^33^S values, leading to the interpretation of deposition from a stratospheric eruptive plume (41). However, in contrast to previous studies that have inferred that the (albeit asymmetric) bipolar distribution of sulfate aerosols associated with the 1453 CE eruption requires a tropical volcano (4, 41), our δ^34^S and Δ^33^S measurements from Greenland suggest that the 1453 CE eruption was a NH extratropical eruption, in agreement with the hypothesis of ref. 50. The initial sulfate deposited in 1453 CE has a Δ^33^S = 0 and low values of δ^34^S (Fig. 1 *H* and *I*), consistent with volcanic sulfur that was transported below the ozone layer, and very similar to the pattern from the 536 CE eruption (this study, Fig. 1 *C* and *D* and *SI Appendix*, Figs. S2 and S3) and the 1912 CE eruption of Katmai/Novarupta or the 1628 BCE eruption of Aniakchak II, both in Alaska (23, 51). In contrast, the isotope signatures for the event in 1458 CE in Greenland and the large event in Antarctica plot along the same Δ^33^S-δ^34^S line as other known tropical eruptions [(e.g., refs. 20, 23, and 32); *SI Appendix*, Fig. S3]. This finding supports the updated alignment of the ice core chronologies (34, 48) that attribute the event in 1458 CE to be a large tropical eruption, likely from the Southern Hemisphere given the large sulfur flux to Antarctica relative to Greenland (50). The chronologies are further verified by the excellent agreement in the timing and magnitude of the isotope excursions between Greenland and Antarctica for the 1458 CE event (Fig. 1*I*), supporting the inference that the sulfate in both hemispheres came from the same eruption. With the Greenland ice core record recently tied with tephra geochemistry to the historic Icelandic 1477 CE Veiðivötn eruption (50), these results firmly anchor the absolute ages of these eruptions to 1453 and 1458 CE.

### Huaynaputina Eruption in 1600 CE.

The large NH summer cooling in 1601 CE has long been attributed to the eruption of Huaynaputina in February 1600 CE (26, 43). Although the geological evidence, based on proximal deposits from this eruption, suggests a very large event (Volcanic Explosivity Index 6), and petrological estimates of sulfur loading indicate that substantial sulfur was released to the atmosphere (26 to 55 Tg S) (52), there remain some questions around the nature of the volcanic forcing at this time. For instance, the forcing associated with the Huaynaputina eruption has a strange hemispheric asymmetry: the NH forcing determined from sulfate flux to Greenland is twice that of the SH forcing despite the volcano being located at 16°S. While it may not be impossible for sulfate aerosols from tropical eruptions to concentrate in the opposite hemisphere (36), the hemispheric asymmetry of the Huaynaputina eruption is a notable outlier in the aerosol distribution and forcings of identified eruptions over the past 2000 y (30).

Previous research has hypothesized the presence of another, unidentified eruption around this time (26). The evidence for this additional eruption comes from high-resolution sulfate measurements in Greenland ice cores which show two distinct peaks in sulfate, as well as tephra found in the Greenland ice core with geochemistry that does not match that of the 1600 CE Huaynaputina eruption (26).

Our sulfur isotopes from Greenland over this time period show that the initially deposited sulfate (e.g., the first sample with sulfate concentrations elevated above background) was likely from an extratropical NH eruption because the sulfate had Δ^33^S values within error of 0‰ and relatively low δ^34^S values, implying transport below the ozone layer, and thus ruling out Huaynaputina due to its 16°S location (Fig. 1 *M* and *N*). These isotope results are supported by tephra shards found in Greenland associated with this first sulfate peak that do not match the geochemistry of Huaynaputina (26). The presence of nonzero Δ^33^S (albeit muted, with values <0.5‰) within this first sulfate peak suggests the plume from this NH extratropical eruption reached the stratosphere (Fig. 1*N*). Sulfate concentrations after this first sulfate peak do not return to background levels before there is another increase in sulfate concentration (Fig. 1*L*), which complicates the pattern of the isotope signatures as the initial samples in the second peak reflect a combination of sulfate from both events. However, this second sulfate peak also included stratospheric sulfate because it has nonzero Δ^33^S throughout the peak (Fig. 1*N*), and thus is a likely candidate for the eruption of Huaynaputina in February 1600 CE. Since the initial increase in sulfate concentration above background levels has been used as an age marker for Huaynaputina for ice core chronologies, an increase in sulfate due to an earlier NH eruption would complicate this attribution. If the second sulfate peak is used as the Huaynaputina tie point based on the conclusions from the isotope results presented here, then the age model around this time should be shifted earlier by one year, which is within the stated uncertainties of the age models (*SI Appendix*, Fig. S4). The relative timing of the initial increase in sulfate for the first eruption relative to seasonally varying aerosols such as Cl and Ca then constrains the timing of the extratropical NH eruption to late 1599/early 1600 CE (*SI Appendix*, Fig. S4). Thus, the extremely cold temperatures reconstructed in 1601 CE should be associated with two eruptions, the 1600 CE eruption of Huaynaputina and an unidentified NH extratropical eruption in winter 1599/1600 CE.

### Revised Forcings for Extratropical Eruptions.

**Fig. 2.**
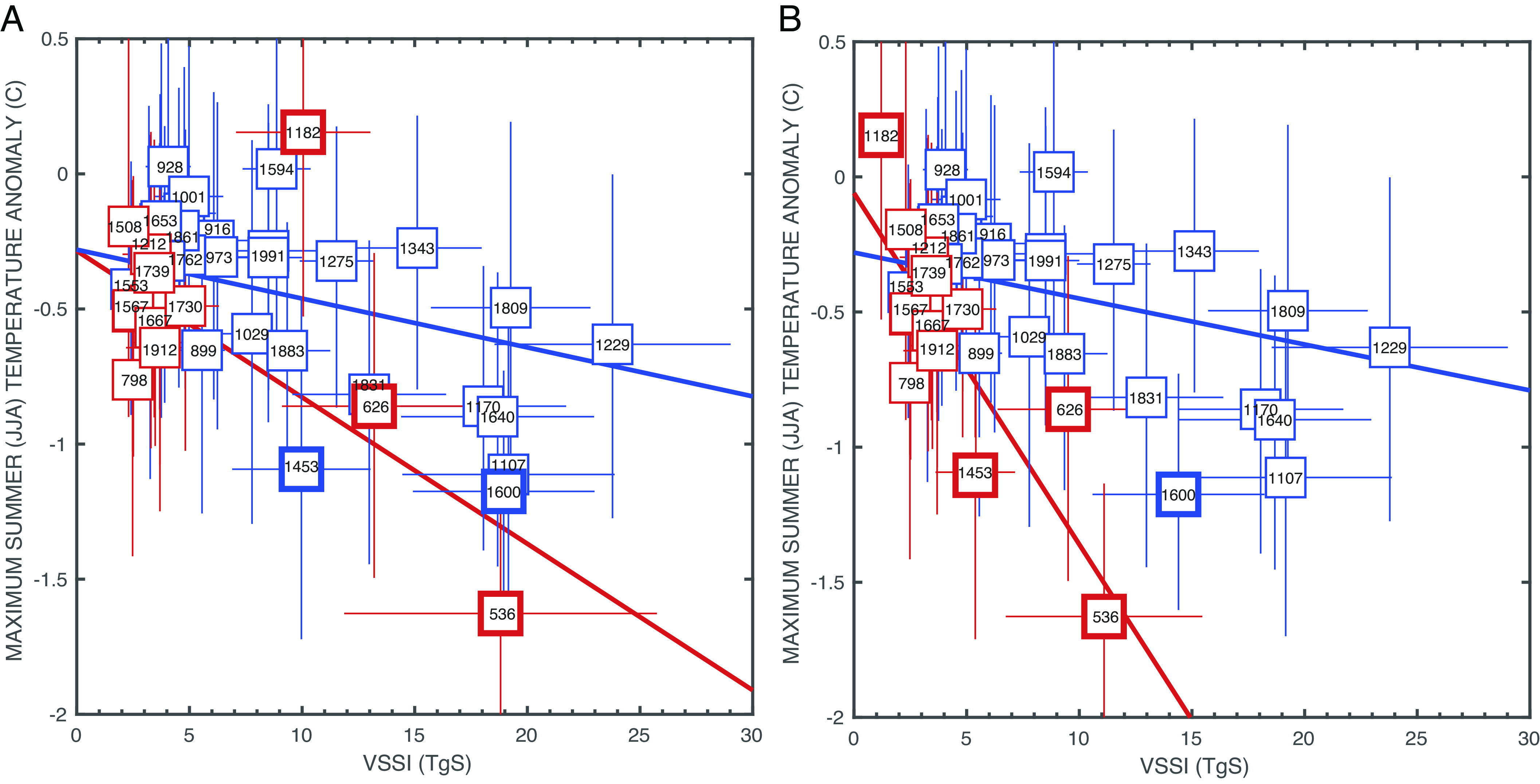
After Toohey et al. (7). (*A*) Maximum NH summer cooling in the years following a volcanic eruption as reconstructed from tree-rings (10, 11, 33) plotted against the stratospheric sulfur loading for that volcanic event [VSSI, (12)] based on sulfate concentrations in polar ice cores assuming all sulfate is stratospheric. Tropical eruptions are plotted in blue [Samalas eruption (1257 CE) plots outside bounds of the chosen limits of graph at 59 Tg S but is plotted in *SI Appendix*, Fig. S9], and extratropical eruptions are plotted in red, based on bipolar and unipolar classification from refs. 4 and 12. (*B*) Same as (*A*) but with the VSSI forcing for the 536, 626, 1182, 1453, and 1600 CE events based on the sulfur isotopes and fraction of stratospheric sulfur determined in this study. The 1453 CE event is plotted here as an extratropical eruption based on the isotope records from this study. Following Toohey et al. (7), no volcanic events are plotted that were immediately preceded (within 6 y) by an additional eruption, with the exception of Huaynaputina (1600 CE) which is preceded by a tropical eruption in 1594, so the temperature response might be impacted by that eruption. Also excluded are known Icelandic eruptions (such as Laki in 1783 CE) because of their proximity to Greenland.

Records of sulfate concentration from polar ice cores have long been used to generate volcanic forcing records for climate model simulations over the CE (3, 12, 53–55), and indeed, they form the basis of the volcanic forcing for the Paleo Model Intercomparison Project 4 (PMIP4) simulations of the last 1000 y [*past1000;* (56)]. Our sulfur isotope data highlight that some of the strongest NH cooling events of the past 2000 y have been associated with extratropical rather than tropical eruptions. For instance, the NH cooling associated with the tropical events of 540 and 1458 CE is less pronounced than the NH cooling associated with the preceding extratropical eruptions in 536 and 1453 CE. This challenges the general view that tropical eruptions have the biggest climatic impact due to the global distribution of aerosols (4). The disproportionate effect of extratropical eruptions on NH summer temperatures also was suggested by Toohey et al. (7), and our data strengthen this inference, for instance, by the inclusion of 1453 CE as an extratropical eruption (Fig. 2).

Moreover, the climatic impact of these extratropical eruptions is even more pronounced and surprising because our sulfur isotope data show that much of the sulfate deposited in these peaks came from below the ozone layer, where aerosol residence times are shorter (7). To further examine the forcing for these events, we combine the high-resolution sulfur isotopes measured over these eruptions with isotope mass balance constraints (following ref. 23; see *Materials and Methods*) to calculate the fraction of sulfate deposited for each of these events that came via the stratospheric overworld (at or above the ozone layer). Integrated across each peak, we find that only 59 ± 8% (1σ) of the sulfate deposited after the 536 CE event and 54 ± 6% (1σ) of the sulfate deposited after the 1453 CE event came from the stratospheric overworld. For the two events at 1600 CE, 76 ± 12% (1σ) of the sulfate was from the stratospheric overworld (Fig. 1 *E*, *J*, and *O*). We also measured sulfur isotopes over the large sulfate peaks in Greenland associated with eruptions in 626 CE and 1182 CE (*SI Appendix*, Fig. S5), confirming their hypothesized NH extratropical source (4, 57). We found that stratospheric overworld sulfate made up only 72 ± 8% and 12 ± 4% (1σ) of the sulfate peaks in 626 and 1182 CE, respectively (*SI Appendix*, Fig. S5 *E* and *J*). Current volcanic forcing records used for model simulations over the CE (e.g., ref. 56) assume that all of the sulfate deposited on polar ice sheets is simply “stratospheric” with aerosol properties for volcanic forcing records tuned to the 1991 eruption of Pinatubo (3, 12). Our isotope results suggest that up to half of the sulfate deposited in Greenland from these NH extratropical eruptions came from the troposphere or LMS, and thus likely had a shorter residence time than assumed in the currently used forcing records. That these NH extratropical eruptions are associated with extreme cooling in NH summer points to a hitherto underappreciated role of short-lived volcanic tropospheric/LMS aerosols in driving NH cooling and/or an even stronger climate sensitivity to volcanic stratospheric overworld aerosols for extratropical eruptions than previously thought [Fig. 2; (7)]. Thus, a key finding of this work is that the currently used volcanic forcings are both overestimating the sulfur loading in the stratospheric overworld and underestimating the sulfur loading in the troposphere/LMS for major extratropical volcanic eruptions.

Although the data presented here mainly come from a single core in Greenland (Tunu2013), we argue that this reduction in the stratospheric overworld sulfate proportion is not a unique feature of this core site. First, we have replicated the sulfur isotopes for the 536 CE event using a second Greenland core (NEEM-2011-S1, *SI Appendix*, Fig. S6), and the mass balance agrees with the results for that event from the Tunu2013 core, with only 50 ± 8% (1σ) of the sulfate deposited coming from the stratospheric overworld [within error of 59 ± 8% (1σ) from Tunu2013]. Furthermore, the relationship between the deposition of sulfate in Tunu2013 versus the mean deposition over Greenland [as calculated from sulfate records from GISP2 (53), NEEM-2011-S1 (34), and NGRIP1 (48)] is similar and within error for tropical (regression slope = 1.04 ± 0.15, 1σ) and extratropical eruptions (regression slope = 1.13 ± 0.08, 1σ), suggesting that the Tunu2013 site is a representative location and does not exhibit a bias for nonstratospheric sulfur deposition for extratropical events (*SI Appendix*, Fig. S7). Additionally, none of these three volcanic events in Tunu2013 (536, 1453, and 1600 CE) show up as having anomalously high sulfate fluxes compared to the Greenland mean (*SI Appendix*, Fig. S7). Finally, continuous sulfur concentration data (calculated from sulfate for consistency) from the higher snow accumulation NGRIP1 core show two distinct sulfur peaks across the years 1600 to 1602 CE (*SI Appendix*, Fig. S8). The first of these two peaks is the NH extratropical eruption and is the smaller of the two events, with an integrated volcanic deposition of sulfate of 12.1 kg/km^2^. The second of these two peaks, which we have attributed to Huaynaputina, has an integrated volcanic deposition of 35.9 kg/km^2^, or 74.7% of the combined flux of the two events, in excellent agreement with the estimates from our isotope mass balance calculations.

The enhanced NH cooling associated with extratropical eruptions compared to tropical eruptions is noteworthy, as it has generally been thought that the largest climatic impacts are associated with low latitude eruptions, due to the longer residence time of their aerosols in the stratosphere (e.g., refs. 5, 58, and 59). While the enhanced sensitivity of proxy temperatures to extratropical eruptions has been illustrated before (7), our revised overworld sulfur loadings more than double this enhanced sensitivity (Fig. 2). Previous work suggested that the sensitivity of reconstructed temperature to stratospheric sulfur loading was a factor of 3.2 greater for extratropical eruptions compared to tropical eruptions (7) (Fig. 2*A*), whereas our revised loadings give a sensitivity that is 7.5 times greater (expressed by regression slopes of −0.13 degrees C/TgS compared to −0.017 degrees C/TgS, for extratropical and tropical eruptions respectively, Fig. 2*B*; see *Materials and Methods*). Model simulations (7) suggest that this observation can be partly explained by the fact that the aerosol load from extratropical eruptions is concentrated in the hemisphere of the eruption, and thus the NH tree-ring records show a strong response to the concentrated aerosol load from an NH eruption. However, the enhanced sensitivity of proxy temperatures to extratropical eruptions is greater than can be explained by the larger NH effective radiative forcing alone (7), suggesting that enhanced climate feedbacks are also required.

Consistent with our results, climate models robustly show amplified temperature responses to extratropical versus tropical forcings, partly as a result of latitudinal differences in radiative feedbacks (60–62). For instance, coupled climate model simulations of a tropical eruption with symmetrical forcing (Pinatubo in 1991 CE, 10 TgS) and an eruption with an NH asymmetrical forcing (Santa Maria in 1902 CE, 1.33 TgS) yield a comparable NH temperature response, despite a factor of 7.5 difference in the sulfur loading of these eruptions (63). This factor of 7.5 difference in modeled climatic response to extratropical versus tropical eruptions is similar to that found in our revised proxy-based estimates, and while somewhat circumstantial being based on different eruptions, it nonetheless provides support to both the robustness of our proxy data and this model’s simulation of volcanic climate response.

These results highlight that extratropical volcanic eruptions can have outsized climatic impacts. It is thus essential that policies are designed to deal with the societal impact of and response to relatively small stratospheric eruptions at high latitudes, alongside the Pinatubo-sized tropical eruptions more commonly discussed. The revised overworld sulfate loadings constrained by our data should facilitate further work to diagnose the transient climate sensitivity resulting from volcanic eruptions and its geographic variability, and may in turn allow improved diagnosis and simulation of the key processes involved, likely some combination of extratropical/tropical contrasts in radiative forcing (e.g., ref. 7), radiative feedbacks [e.g., sea ice albedo (64)], and ocean heat uptake (65). Our data highlight the critical importance of the spatial structure of sulfate distribution to associated climate response (e.g., refs. 63 and 66). This has important implications for understanding climatic response to sulfate aerosol forcing more generally, whether volcanic or artificial, underscoring the complexities involved in proposals to geoengineer our way out of harm from anthropogenic climate change.

## Materials and Methods

### Ice Core Samples and S Isotope Measurement.

Ice core samples from the 1600, 1453, and 626 CE events were cut from the Tunu2013 ice core in Greenland (78.04°N, 33.88°W). The volcanic events at 540 CE and 1458 CE were sampled from both the Tunu2013 ice core and the B40 ice core in Antarctica (75.0°S, 0.07°E). The 536 CE event was sampled from both the Tunu2013 and NEEM-2011-S1 (77.45°N, 51.06°W) ice cores in Greenland. The samples from the 1182 event were cut from the NGRIP1 (75.1°N, 42.32°W) ice core in Greenland. In all cases, continuous high-resolution sulfur concentration data (4, 31, 34, 48) were used to identify the peaks associated with volcanic events, and discrete samples were taken across the peak at a resolution between 2 and 4 cm length, corresponding to approximately 3 to 6 samples per year. Additionally, for each event, at least one background sample prior to the start of the volcanic sulfate peak was collected at a resolution of 5 cm, covering approximately 5 mo. The concentrations of the discrete samples were measured by ion chromatography in the St Andrews isotope Geochemistry (STAiG) laboratory at the University of St Andrews with a Metrohm 930 Compact IC Flex to determine how much sample was needed to get 20 nmol of sulfate. The samples were dried down and put through columns following the procedure described by ref. 23. Triple S isotopes (^32^S, ^33^S, and ^34^S) were measured by multicollector inductively coupled plasma mass spectrometry on a Neptune Plus in the STAiG laboratory. Precision and external reproducibility of isotope measurements were monitored with an in-house consistency standard [Switzer Falls: δ^34^S = 4.11 ± 0.12‰ V-CDT (Vienna–Canyon Diablo Troilite), 1 SD, (67)] and over the course of this study gave a measured value of δ^34^S = 4.16 ± 0.06 and Δ^33^S = 0.01 ± 0.05 ‰ (n = 15, 1 SD). With each set of 14 samples, a total procedural blank was processed. The average and SD of all the blanks measured over the course this study (0.14 ± 0.02 nmol, with a δ^34^S of 5.6 ± 0.9‰; n = 10, 1 SD) was used to correct the measured isotope ratios in samples.

### Interpretation of Sulfur Isotopes in Ice Cores.

The sulfur isotope values (δ^34^S and δ^33^S) reported here are relative to the V-CDT standard, and δ^x^S = (^x^S/^32^S)_sample_/(^x^S/^32^S)_VCDT_ − 1. A sample is considered to have a MIF signature if it has a nonzero value of Δ^33^S = δ^33^S − ((δ^34^S + 1)^0.515^ − 1), outside of 2σ uncertainty. To estimate the isotopic composition of the volcanic sulfate in each sample, the contribution of the background sulfate was removed using the concentration and isotope measurements on the background samples associated with that event and equations of isotope mass balance (20, 23, 32). Uncertainties on this correction were propagated using Monte Carlo simulations. Similar to previous studies (23, 32), samples with more than 35% of the sulfate coming from background sources had propagated uncertainties on the isotope ratios that were prohibitively large to interpret and so were not plotted in these figures.

Although the presence of a nonzero Δ^33^S implies that the sulfate came from the stratosphere at altitudes at or above the ozone layer (18), the interpretation of a Δ^33^S = 0 (within uncertainty) is more nuanced. It cannot be strictly interpreted as tropospheric since there is a region of the lower stratosphere in the high latitudes that is below the ozone layer. Thus, sulfur from these altitudes in the lower stratosphere would not inherit a MIF signal (24). However, modeling results suggest that sulfate aerosols that are injected into the LMS at high latitudes are rapidly transported along potential temperature isentropes into the troposphere and thus have a much shorter residence time compared to those in the upper stratosphere at or above the ozone layer in the stratospheric overworld (7). As such, ice core sulfate Δ^33^S still identifies sulfate that had a long residence time in the stratosphere and thus a large climate forcing potential.

We use the slope of Δ^33^S v δ^34^S in ice core volcanic sulfate (*SI Appendix*, Figs. S2 and S3) to distinguish between eruptions that came from the tropics and those that came from the extratropics (23). Photochemical reactions in the stratospheric overworld impart a distinctive pattern of Δ^33^S v δ^34^S with a slope of 0.09 (20, 23, 32). Since all of the sulfate deposited on the polar ice sheets from tropical eruptions comes via the stratospheric overworld, this signature is preserved. In contrast, if sulfate from extratropical eruptions is deposited on the ice sheet via the LMS or the troposphere, then its isotopic signature across the sulfate peak will reflect a time-varying mixture between 1) mass dependently fractionated sulfate with a Δ^33^S = 0 and a δ^34^S reflecting the isotopic signature of the erupted sulfur and 2) any stratospheric overworld sulfate with a Δ^33^S v δ^34^S slope of 0.09 (23).

### Isotope Mass Balance Calculation.

Following Burke et al. (23), the fraction of sulfate within a sample coming from altitudes in the atmosphere at or above the ozone layer can be solved by isotope mass balance. We use the following two equations to relate the proportions of three types of sulfate from different sources: *f_strat_* is the proportion of sulfate coming from the stratospheric overworld*, f_trop_* is the proportion of sulfate coming from the troposphere or LMS, and *f_bkgd_* is the proportion of sulfate delivered to the ice sheet that is not from the volcanic event in question (of which marine biogenic sulfur is a major part).

[1] $$\delta^{34}S_{\mathrm{meas}}=\delta^{34}S_{\mathrm{strat}}f_{\mathrm{strat}}+\delta^{34}S_{\mathrm{trop}}\left(1-f_{\mathrm{strat}}-f_{\mathrm{bkgd}}\right)+\delta^{34}S_{\mathrm{bkgd}}f_{\mathrm{bkgd}},$$

[2] $$\delta^{33}S_{\mathrm{meas}}=\delta^{33}S_{\mathrm{strat}}f_{\mathrm{strat}}+\delta^{33}S_{\mathrm{trop}}\left(1-f_{\mathrm{strat}}-f_{\mathrm{bkgd}}\right)+\delta^{33}S_{\mathrm{bkgd}}f_{\mathrm{bkgd}}.$$

By definition, these fractions (*f_strat_*, *f_trop_*, *f_bkgd_*) must be between 0 and 1, and their sum must equal 1. Furthermore, to reduce the number of unknowns, we can use constraints from the terrestrial mass-dependent fractionation line and the fractionation line reconstructed from stratospheric sulfate from tropical eruptions (20, 23, 32) to relate the δ^33^S to the δ^34^S values as follows:

[3] $$\delta^{33}S_{\text{trop}}=\left({\left(\delta^{34}S_{\text{trop}}+1\right)}^{0.515}-1\right),$$

[4] $$\delta^{33}S_{\text{bkgd}}=\left({\left(\delta^{34}S_{\text{bkgd}}+1\right)}^{0.515}-1\right),$$

[5] $$\delta^{33}S_{\text{strat}}=\left({\left(\delta^{34}S_{\text{strat}}+1\right)}^{0.608}-1\right).$$

Substituting Eqs. **3**–**5** into Eqs. **1** and **2** yields two equations with three unknowns. By making an assumption about the δ^34^S value of the sulfur erupted from the volcano (δ^34^S_trop_) prior to any fractionation by ultraviolet radiation, a solution can be found numerically for f_strat_ and Δ^34^S_strat_. Since the δ^34^S_trop_ can vary widely as shown in a recent compilation by ref. 68, we calculate the fraction of stratospheric sulfate and associated uncertainty using 10,000 Monte Carlo simulations that assume a uniform distribution of the initial δ^34^S between −5 and 10‰, and normally distributed uncertainties on the measured δ^34^S and δ^33^S values and the value of the exponent (λ = 0.608 ± 0.006) that relates δ^34^S and δ^33^S values in stratospheric sulfate (23). For a given eruption, each Monte Carlo simulation samples a value of λ from these prior distributions described above, and these values are carried through all samples for that eruption. We restrict solutions to satisfy the conditions of -60‰ < δ^34^S_strat_ < 30‰, which is based on previously published stratospheric sulfate values from tropical eruptions (20, 23, 32). We also require that the initial sample of an eruption (the deepest in core) that has a nonzero MIF signal (outside of 2σ measurement uncertainty) must be positive, in line with the evolution of stratospheric sulfate isotopes in previous work (e.g., refs. 19, 22, 23, and 32), with a further final constraint that each subsequent sample in a given eruption needs to have a δ^34^S_strat_ that is less than the previous sample. This final constraint is relaxed for the case of Huaynaputina where two back-to-back eruptions could result in going from a more negative to a more positive δ^34^S_strat_ across the two sulfate peaks. Any samples at the start of the sulfate peak that have a Δ^33^S value within 2σ uncertainty of 0 (−0.1 < Δ^33^S < 0.1) are interpreted to have no stratospheric sulfate. Matlab software to calculate these stratospheric fractions is available on our GitHub repository: https://github.com/St-Andrews-Isotope-Geochemistry/Burke_2023_PNAS (69).

### Tree-Ring Temperature Anomalies and Temperature Sensitivities to Volcanic Forcing.

Four different tree-ring reconstructions were used in estimating the temperature anomaly following an eruption: Wil16 (11), Sch15 (9), Sto15 (10), and Gui17 (33). Tree-ring temperature anomalies were calculated relative to the temperature estimates in the 3 y prior to the eruption year for all the eruptions considered in ref. 7, which excluded known effusive Icelandic eruptions and eruptions for which the background NH temperature may have been influenced by an earlier eruption. The maximum temperature anomalies plotted in Fig. 2 are the coldest temperature anomalies in the 2 y following the eruption (inclusive of the eruption year). We take this approach because there is an inherent age uncertainty in the forcing/cooling relationship of up to 1 y arising from the unknown seasonal timing of the eruption and the fact that the trees used in the temperature reconstructions only record summer temperatures. Furthermore, there is an additional ice core age uncertainty on the order of ±1 y for most eruptions [although it is zero years uncertainty for eruptions in 536 CE, 540 CE, 1453 CE, and 1458 CE because of additional age constraints provided through historic observations of volcanic dust veils, i.e., in March 536 CE in Europe and in February 1477 CE in Iceland following the Veiðivötn eruption (4, 50)]. For each eruption, the maximum anomaly was found for each of the 4 tree-ring reconstructions [Wil16 (11), Sch15 (9), Sto15 (10), and Gui17 (33)], and these four anomalies were averaged and plotted in Fig. 2.

Maximum likelihood regressions were determined on the tropical and extratropical datasets taking into account uncertainties in both x and y variables (stratospheric sulfur loading and temperature anomaly) following (70). If the original stratospheric sulfur estimates and tropical/extratropical attributions are used (Fig. 2*A*; 12), then the tropical regression slope is −0.018 degrees/TgS (95% CI: −0.042 to −0.01 degrees/TgS; r^2^ = 0.30), whereas the extratropical slope is −0.054 degrees/TgS (95% CI: −0.074 to 0.048 degrees/TgS; r^2^ = 0.34). However, when the revised sulfur loadings and tropical/extratropical attributions are used (Fig. 2*B*; this study), the slope for tropical eruptions remains similar with a value of −0.017 degrees/TgS (95% CI: −0.041 to −0.0099 degrees/TgS; r^2^ = 0.30), but the extratropical slope is much larger at −0.13 degrees/TgS (95% CI: −0.22 to −0.06 degrees/TgS; r^2^ = 0.68). The inclusion of the 1257 CE eruption of Samalas [VSSI (Volcanic stratospheric sulfur injection) = 59.42 Tg] exerts a large influence on the regression given the large size of the sulfur loading relative to the other eruptions. Excluding this eruption from the regression increases the sensitivity of tropical eruptions to −0.037 degrees/TgS (95% CI: −0.052 to −0.021 degrees/TgS; r^2^ = 0.41), but the slope is still outside the 95% CI from the extratropical eruptions (*SI Appendix*, Fig. S9).

The same calculation can be done, but instead of using the maximum cooling in the 2 y following the eruption, the mean of the 2 y following the eruption is used. The results from this analysis can be seen in *SI Appendix*, Fig. S10. In this case, when the revised sulfur loadings and tropical/extratropical attributions are used, the slope for tropical eruptions has a value of −0.011 degrees/TgS (95% CI: −0.03 to −0.006 degrees/TgS; r^2^ = 0.24), but the extratropical slope is still larger at −0.1 degrees/TgS (95% CI: −0.17 to −0.03 degrees/TgS; r^2^ = 0.62). Again, these slopes are different at the 95% CI.

## Supplementary Material

Appendix 01 (PDF)

Dataset S01 (XLSX)

## Data Availability

Matlab software to calculate stratospheric fraction of sulfate using ice core sulfur isotope data can be accessed on GitHub (https://github.com/St-Andrews-Isotope-Geochemistry/Burke_2023_PNAS) (69). All other data are included in the manuscript and/or supporting information.
