# Supplementary material for: High sensitivity of summer temperatures to stratospheric sulfur loading from volcanoes in the Northern Hemisphere
Source: Proc Natl Acad Sci U S A. 2023 Nov 6;120(47):e2221810120. doi: 10.1073/pnas.2221810120 (PMC10666123; doi:10.1073/pnas.2221810120)
Supplement: Supplementary file 1 — Appendix 01 (PDF) [file pnas.2221810120.sapp.pdf]

PNAS

Supporting Information for

High sensitivity of summer temperatures to stratospheric sulfur loading from volcanoes in the Northern Hemisphere

Andrea Burke[a], Helen M. Innes[a], Laura Crick[a], Kevin J. Anchukaitis[b], Michael P. Byrne[a], William Hutchison[a], Joseph R. McConnell[c], Kathryn A. Moore[a,d], James W.B. Rae[a], Michael Sigl[e], and Rob Wilson[a]

[a]School of Earth and Environmental Sciences, University of St Andrews, St Andrews, KY16 9TS, United Kingdom
[b]School of Geography, Development and Environment and Laboratory of Tree-Ring Research, University of Arizona, Tucson, AZ 85721, USA
[c]Division of Hydrologic Sciences, Desert Research Institute, Reno, NV 89512, USA
[d]Department of Atmospheric Science, Colorado State University, Fort Collins, CO, 80523, USA
[e]Climate and Environmental Physics & Oeschger Centre for Climate Change Research, Universtity of Bern, 3012 Bern, Switzerland

Corresponding author: Andrea Burke
Email: ab276@st-andrews.ac.uk

This PDF file includes:

Figures S1 to S10
Tables S1
SI References

Other supporting materials for this manuscript include the following:

Dataset

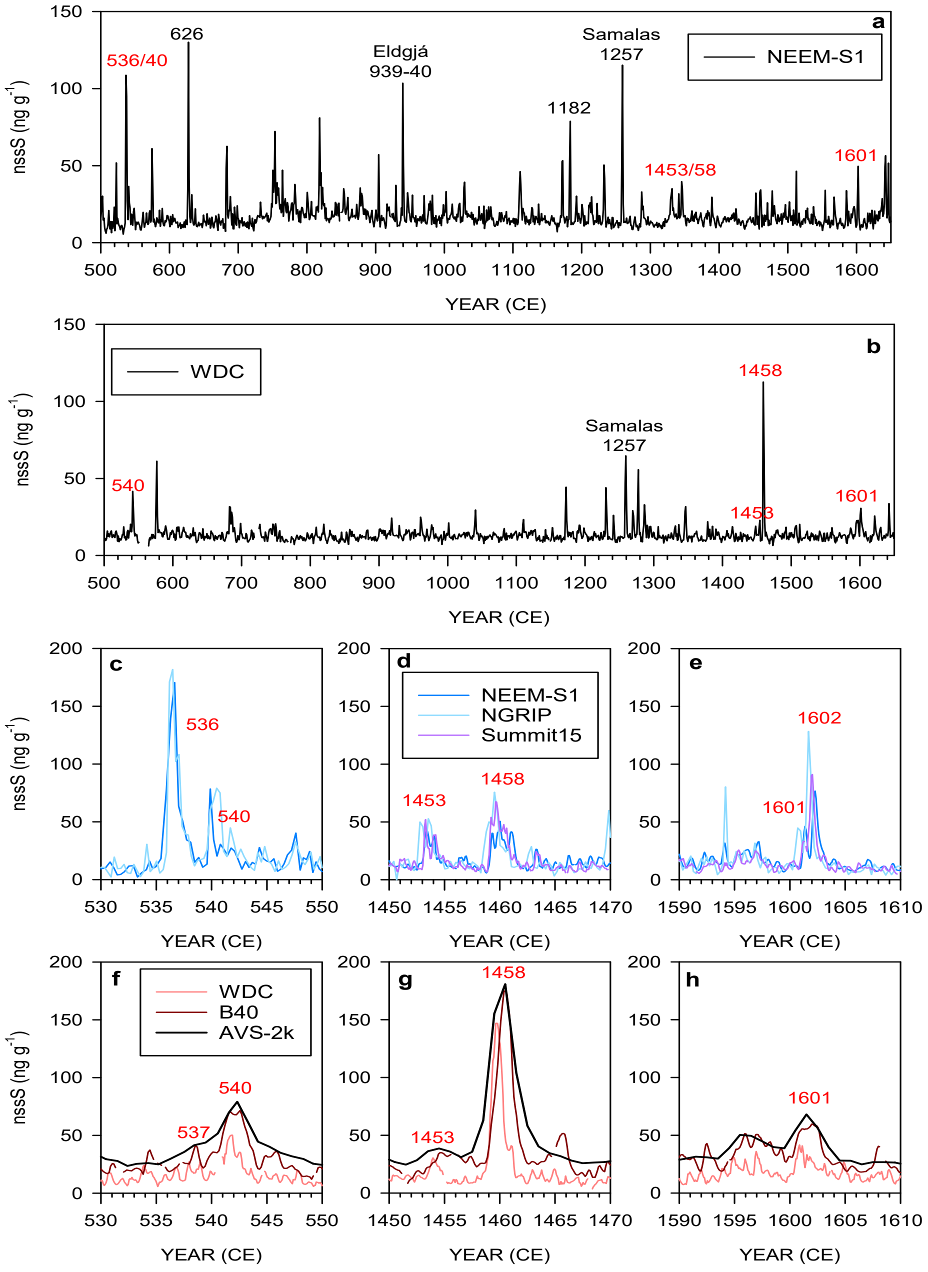

Fig. S1. Continuous ice core records of annual mean non-sea salt (nss) sulfur concentration for the time period 500 to 1650 CE from a) Greenland (NEEM-S1; **(1)**) and b) Antarctica (WDC; **(1)**) on the NS1-2011 and WD2014 chronologies **(2, 3)**); key eruptions are indicated including the "double events" (red) analyzed for sulfur isotopes; c)-e) high time resolution nss-sulfur concentrations for Greenland ice cores NEEM-S1 **(1)**, NGRIP **(4)** and Summit15 **(5)** for the time windows 530-550 CE, 1450-1470 CE and 1590-1610 CE; f)-h) high time resolution nss-sulfur concentrations for Antarctica ice cores WDC **(1)**, B40 **(6)**, and a stack (12≤N≤14) of synchronized ice cores from Antarctica (AVS2k; **(6)**) for the time windows 530-550 CE, 1450-1470 CE and 1590-1610 CE.

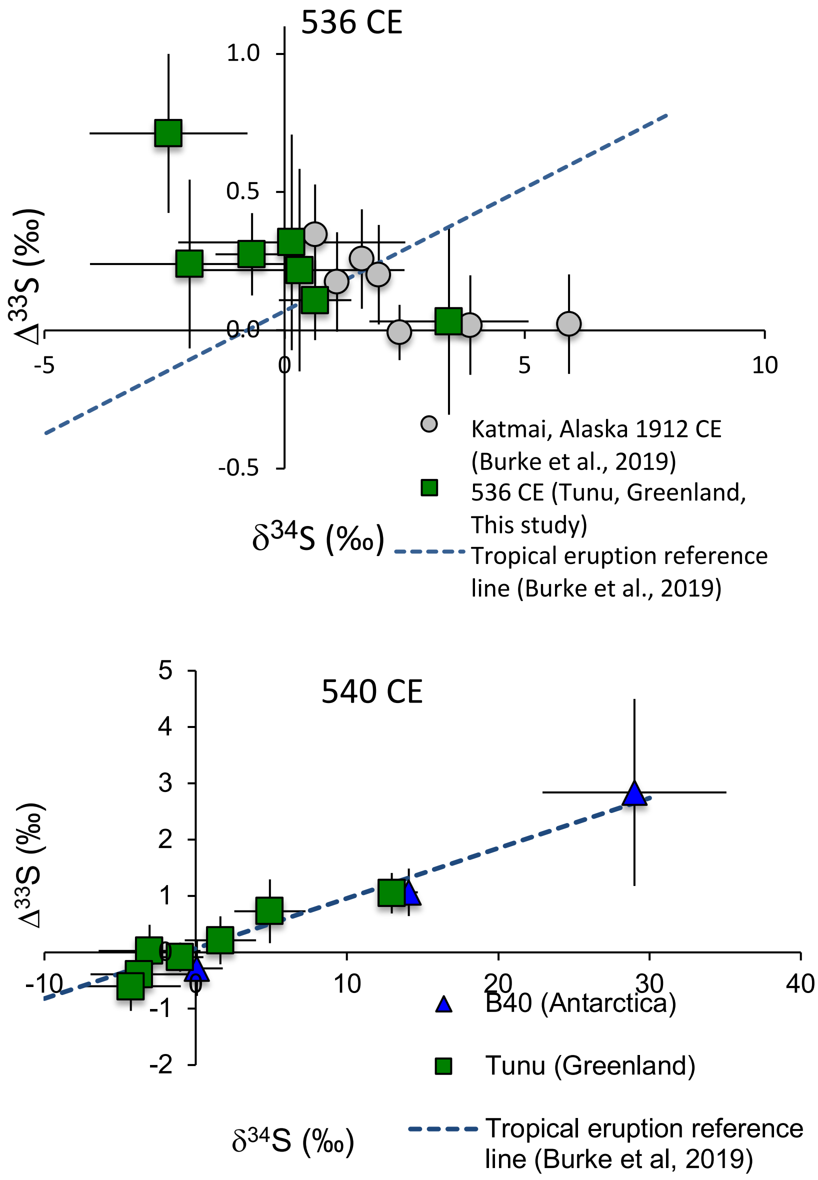

Fig. S2. $\Delta^{33}S$(‰, V-CDT) versus $\delta^{34}S$ (‰, V-CDT) of volcanic sulfate in the Tunu2013 Greenland ice core (green squares) and the B-40 Antarctic ice cores (blue triangles) for the eruption at 536 CE (top) and 540 CE (bottom). For reference, the relationship between $\Delta^{33}S$ and $\delta^{34}S$ for known tropical eruptions (Tambora and Samalas; **(7)**) is plotted as a dashed line in both panels. The measured $\Delta^{33}S$ and $\delta^{34}S$ from the extratropical eruption of Katmai/Novarupta in 1912 from the Tunu2013 core in Greenland is also shown in grey circles in the top panel for reference **(7)**. Extratropical eruptions have a different slope from the tropical stratospheric reference line because the isotopes represent a varying mixture of stratospheric sulfate and non-MIF mass dependent sulfate. Only samples from 536 and 540 CE that had less than 35% of their sulfate from background sources are plotted for clarity. Uncertainty bars represent 1 σ uncertainty after Monte Carlo propagation of uncertainty of the background correction. Measurement uncertainty of $\delta^{34}S$ and $\Delta^{33}S$ is much smaller and would be smaller than the symbol size (~0.1‰ for both $\delta^{34}S$ and $\Delta^{33}S$)

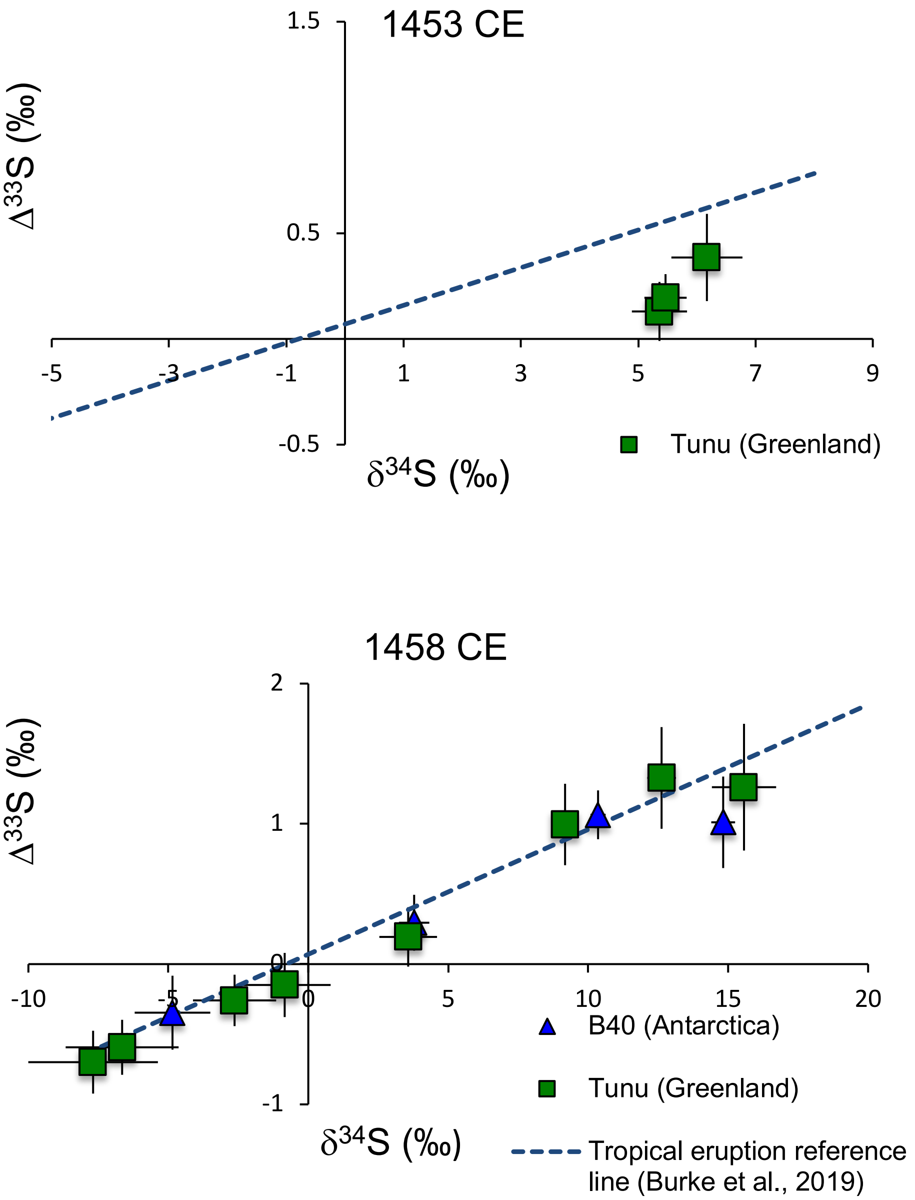

Fig. S3. $\Delta^{33}S$(‰, V-CDT) versus $\delta^{34}S$ (‰, V-CDT) of volcanic sulfate in the Tunu2013 Greenland ice core (green squares) and the B-40 Antarctic ice cores (blue triangles) for the eruption at 1453 CE (top) and 1458 CE (bottom). For reference, the relationship between $\Delta^{33}S$ and $\delta^{34}S$ for known tropical eruptions (Tambora and Samalas; (7)) is plotted as a dashed line. Extratropical eruptions have a different slope from the tropical stratospheric reference line because the isotopes represent a varying mixture of stratospheric sulfate and non-MIF mass dependent sulfate. Only samples that had less than 35% of their sulfate from background sources are plotted for clarity. Uncertainty bars represent 1 σ uncertainty after Monte Carlo propagation of uncertainty of the background correction. Measurement uncertainty of $\delta^{34}S$ and $\Delta^{33}S$ is much smaller and would be smaller than the symbol size (~0.1‰ for both $\delta^{34}S$ and $\Delta^{33}S$)

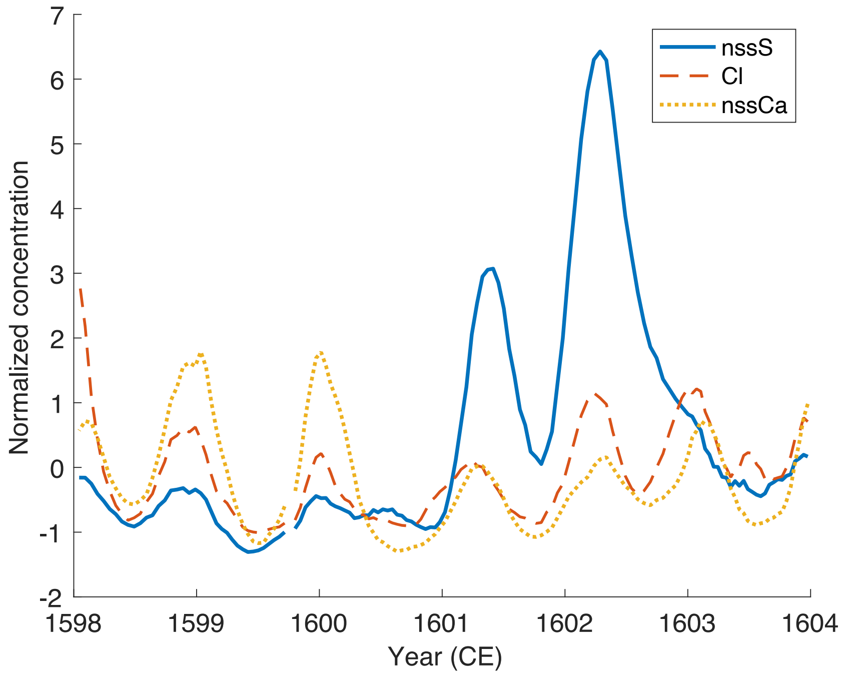

Fig. S4. Seasonality of the eruptions in the early 1600s CE. Normalized concentration data of non-sea salt sulfur (nssS; blue line), chlorine (Cl; red dashed line), and non-sea salt calcium (nssCa; yellow dotted line) from the NEEM_2011_S1 ice core **(2)**. The normalization process removed long-term trends and normalized the residuals to have a mean of 0 and a standard deviation of 1. The data is plotted on the published age model **(2)**. Close inspection of the seasonally varying aerosols Cl (peak in mid winter) and Ca (peak in winter/spring) suggests that the initial increase in sulfate as part of the double event attributed to Huaynaputina occurred prior to the peak in Cl, putting its timing in late 1600 CE. The arrival of the second peak in sulfate occurs in the summer of 1601 CE. To make this timing consistent with the timing of the eruption of Huaynaputina in February 1600 CE, the age model would need to be shifted earlier by one year.

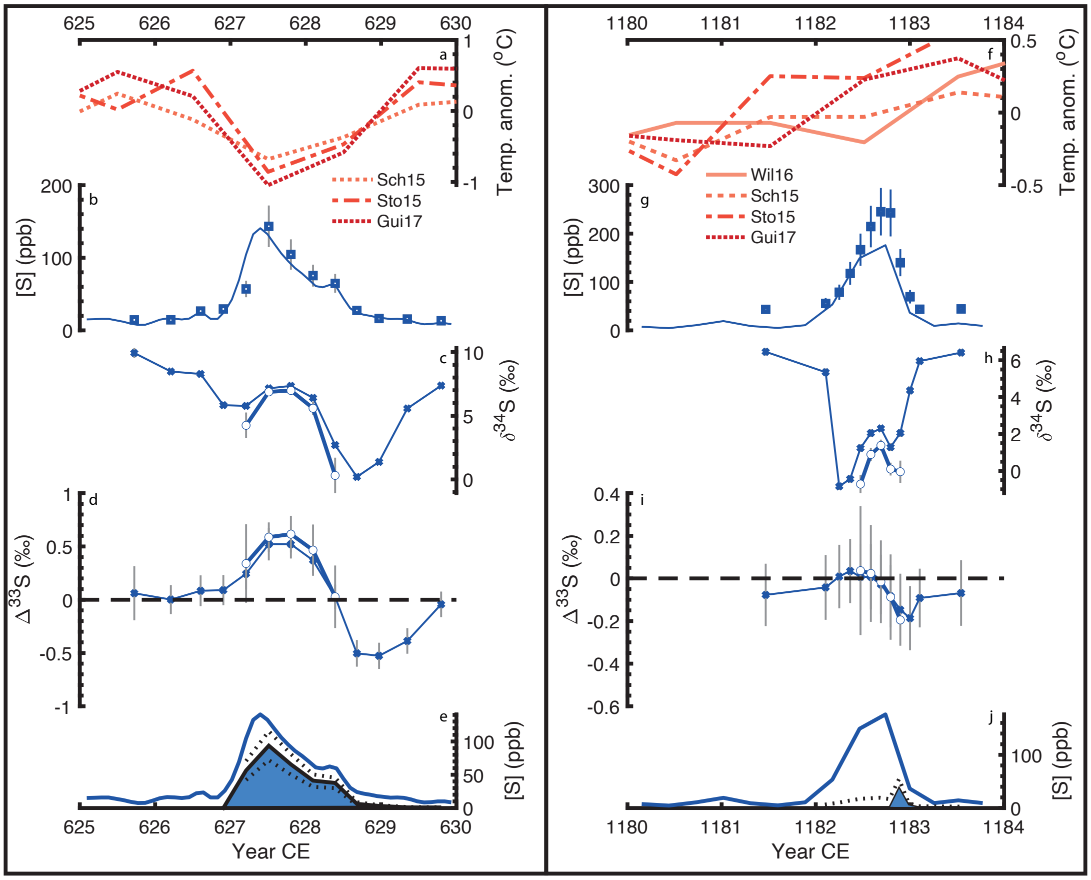

Fig. S5. Sulfur concentration and isotope results for the volcanic event at 626 CE in the Tunu2013 ice core (left panel, **a-e**) and 1182 CE in the NGRIP1 ice core (right panel, **f-j**). **(a,f)** Northern Hemisphere summer temperature anomaly relative to the three year mean prior to the eruption reconstructed from tree-rings (Wil16 (8), Sch15(9), Sto15(10), Gui17 (11)). **(b,g)** Concentration of sulfur (ppb), where the line is from continuous measurement on the ice core (2, 4) and squares are the discrete concentration measurements made on the isotope samples. **(c,h)** $\delta^{34}S$ and **(d,i)** $\Delta^{33}S$ (‰, V-CDT) measured in ice core samples (x's) as well as the background corrected $\delta^{34}S$ values for volcanic sulfate (o's) for samples with more than 65% volcanic sulfate **(e,j)** Concentration of stratospheric sulfate solved by isotope mass balance (shaded area, with dashed lines representing 1 σ uncertainty) compared to total sulfate (blue line), which also includes sulfate from background sources and from volcanic sulfate transported below the ozone layer.

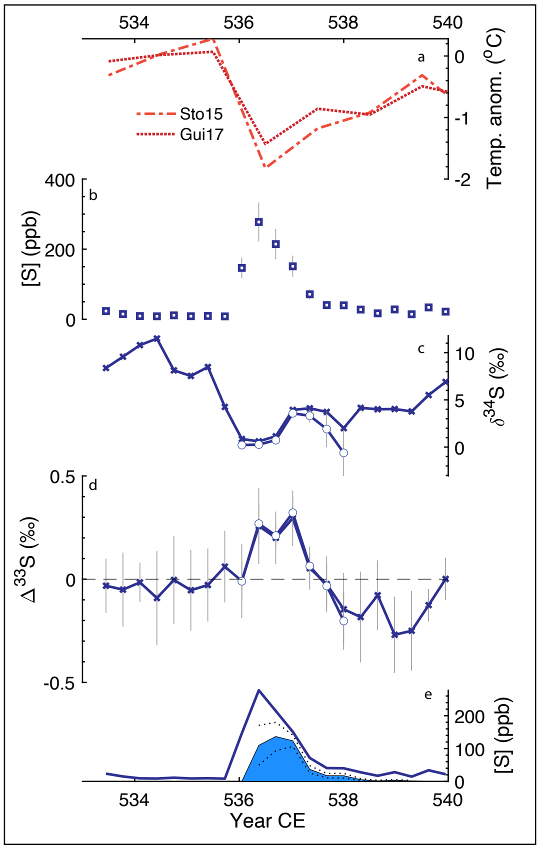

Fig. S6. Sulfur isotope results from the NEEM ice core for the volcanic event at 536 CE. (a) Northern Hemisphere summer temperature anomaly relative to the three year mean prior to the eruption reconstructed from tree-rings (Sto15 (10), Gui17 (11)). (b) Sulfur concentration (ppb) measured in discrete isotope samples. (c) $\delta^{34}S$ and (d) $\Delta^{33}S$ (‰, V-CDT) measured in ice core samples (x's) as well as the background corrected $\delta^{34}S$ values for volcanic sulfate (o's) in ice cores. (e) Concentration of stratospheric sulfate solved by isotope mass balance (shaded area, with dashed lines representing 1 σ uncertainty) compared to total sulfate (blue line), which also includes sulfate from background sources and from volcanic sulfate transported below the ozone layer.

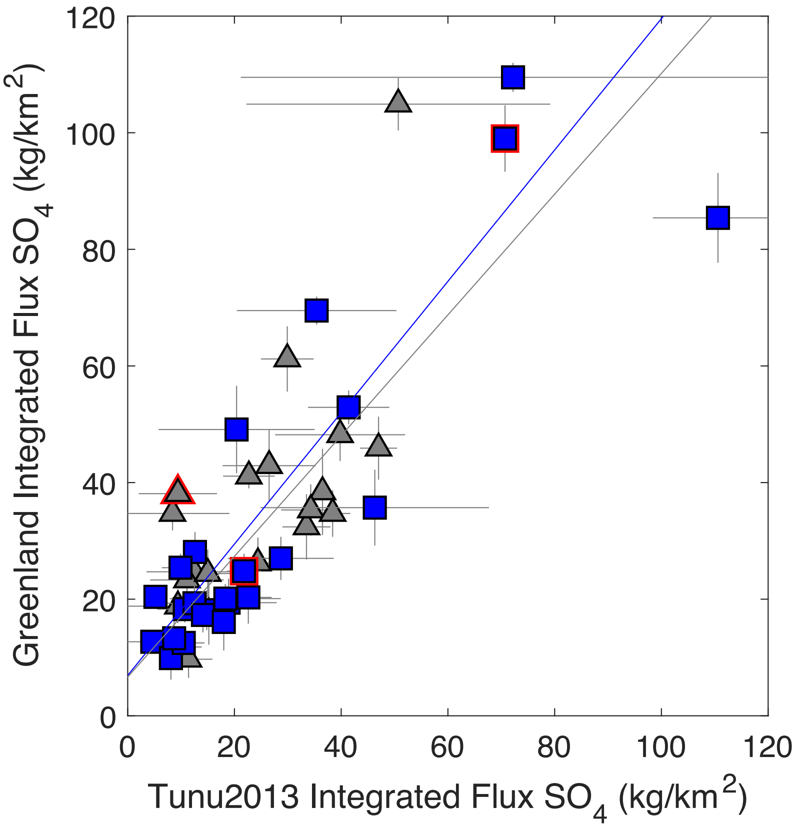

Fig. S7. The integrated flux of sulfate across volcanic peaks in the Tunu2013 ice core, compared with the average integrated flux of sulfate across volcanic events computed from sulfate concentrations in GISP2 (12) , NEEM (1), and NGRIP1 (4) ice cores. Tropical eruptions are grey triangles and extratropical eruptions are blue squares, following attributions in (2), except where updated in this study (e.g. the 1453 CE eruption). Eruptions measured in this study are highlighted in red (triangle: Huaynaputina, square with low flux: 1453 CE, square with high flux: 536 CE). Error bars are 1 σ.

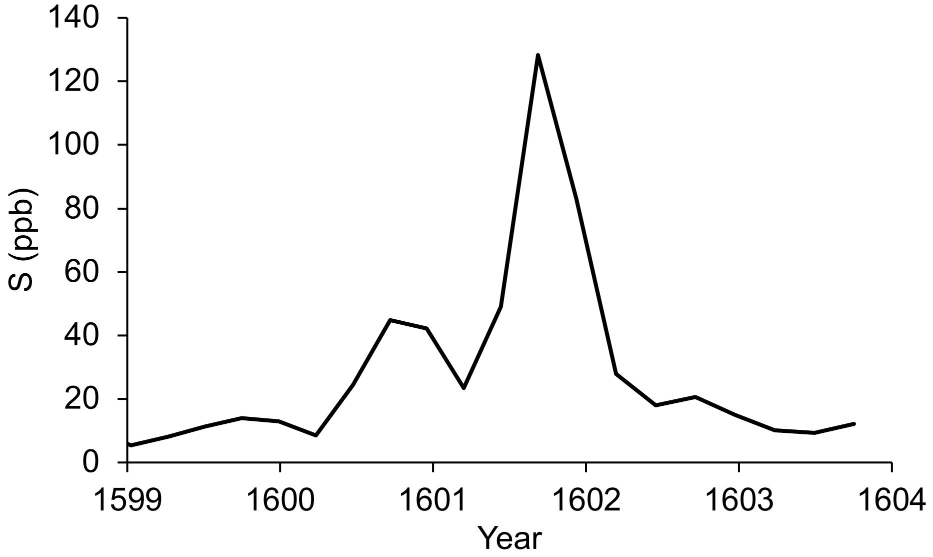

Fig. S8. Sulfur concentration data from NGRIP1 (4). The initial peak arriving in late 1600 CE is a NH extratropical eruption, and the second peak is the Huaynaputina eruption of 1600 CE. Combined, these peaks lead to an integrated volcanic deposition of 48 kg/km^2. The second peak alone has an integrated volcanic deposition of 35.9 kg/km^2, or 74.7% of the combined flux.

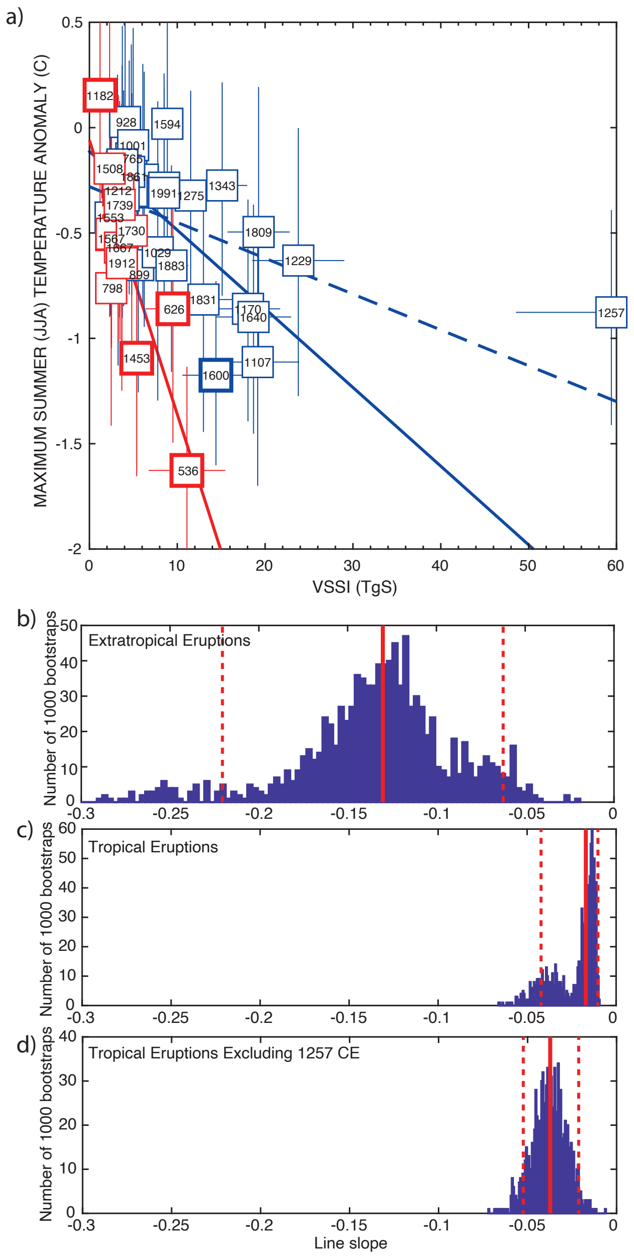

Fig. S9. (a) Same as Fig. 2b but with x-axis expanded to allow 1257 CE eruption (Samalas) to be plotted at 59 Tg S. Dashed blue line is the median maximum likelihood regression for tropical eruptions including the 1257 CE eruption (as in Fig 2b), and solid blue line is the median maximum likelihood regression for tropical eruptions excluding the 1257 CE eruption. Red line is the median maximum likelihood regression for extratropical eruptions (b-d) Histograms of the slopes determined from bootstrapping maximum likelihood regressions on the extratropical eruptions (b), all tropical eruptions (c), and tropical eruptions excluding 1257 CE (d) following the approach of (13) which accounts for uncertainty in both VSSI (Tg S) and temperature anomaly estimates. Vertical red lines show the median of the distribution, and the vertical red dashed lines show the 95% confidence interval.

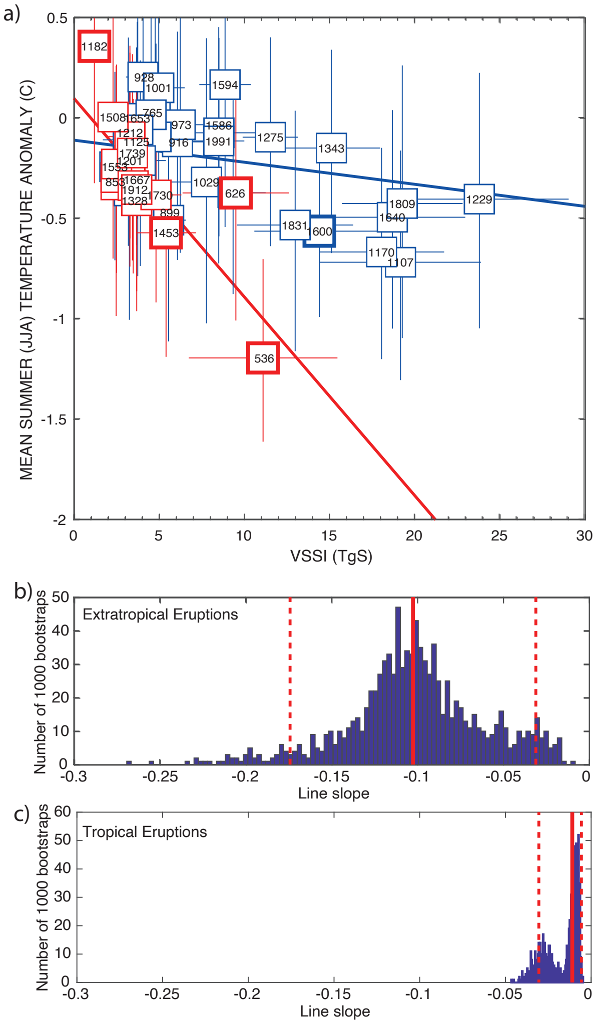

Fig S10. (a) Same as Fig. 2b but with NH temperature anomalies calculated as the mean of the 2 years following the eruption year. Bold lines are based on the median maximum likelihood regressions for tropical (blue) and extratropical (red) eruptions. (b,c) Histograms of the slopes determined from bootstrapping maximum likelihood regressions on the extratropical eruptions (b) and tropical eruptions (c) following the approach of (13) which accounts for uncertainty in both VSSI (Tg S) and temperature anomaly estimates. Vertical red lines show the median of the distribution, and the vertical red dashed lines show the 95% confidence interval.

Table S1. Metadata for volcanic eruptions. Timing, sulfur injection and peak sulfate aerosol optical depth (SAOD) for volcanic eruptions discussed in this study.

Year (CE)	Stratospheric Sulfur Injection (TgS)[a]	Global Radiative Peak Forcing (W m^{-2})	Peak $SAOD_{30\text{-}90°N}$ [a]	Peak $SAOD_{30\text{-}90°S}$ [a]	Suggested provenance [b]
1600	19 (±4)	-4.9	0.247	0.155	Huaynaputina (Peru)
1458	33 (±5)	-7.7	0.273	0.427	Kuwae (Vanuatu)
1453	10 (±3)	-2.7	0.133	0.048	Unidentified
1257	59 (±11)	-11.2	0.580	0.402	Samalas (Indonesia)
1182	10 (±3)	-3.3	0.326	0.053	Katla (Iceland)
626	13 (±4)	-4.3	0.404	0.068	Unidentified
540	32 (±8)	-7.5	0.360	0.281	Unidentified
536	19 (±7)	-5.7	0.511	0.096	Unidentified

[a] From reference (14); [b] From reference (1)

Dataset (separate file). Sulfur concentration and isotope ($\delta^{34}S$, $\delta^{33}S$, and $\Delta^{33}S$) data from ice core samples.

SI References

1. Sigl M, et al. (2013) A new bipolar ice core record of volcanism from WAIS Divide and NEEM and implications for climate forcing of the last 2000 years. *J Geophys Res Atmos* 118(3):1151–1169.

2. Sigl M, et al. (2015) Timing and climate forcing of volcanic eruptions for the past 2,500 years. 523(7562):543–549.

3. Sigl M, et al. (2016) The WAIS Divide deep ice core WD2014 chronology – Part 2: Annual-layer counting (0–31 ka BP). *Clim Past* 12(3):769–786.

4. Plummer CT, et al. (2012) An independently dated 2000-yr volcanic record from Law Dome, East Antarctica, including a new perspective on the dating of the 1450s CE eruption of Kuwae, Vanuatu. *Clim Past* 8(6):1929–1940.

5. Stoffel M, et al. (2022) Climatic, weather, and socio-economic conditions corresponding to the mid-17th-century eruption cluster. *Clim Past* 18(5):1083–1108.

6. Sigl M, et al. (2014) Insights from Antarctica on volcanic forcing during the Common Era. *Nature Clim Change* 4(8):693–697.

7. Burke A, et al. (2019) Stratospheric eruptions from tropical and extra-tropical volcanoes constrained using high-resolution sulfur isotopes in ice cores. *Earth and Planetary Science Letters* 521:113–119.

8. Wilson R, et al. (2016) Last millennium northern hemisphere summer temperatures from tree rings: Part I: The long term context. *Quaternary Science Reviews* 134(C):1–18.

9. Schneider, Lea, et al. (2015) Revising midlatitude summer temperatures back to AD 600 based on a wood density network. *Geophysical Research Letters* 42: 4556-4562.

10. Stoffel M, et al. (2015) Estimates of volcanic-induced cooling in the Northern Hemisphere over the past 1,500 years. *Nature Geoscience* 8(10):784–788.

11. Guillet S, et al. (2017) Climate response to the Samalas volcanic eruption in 1257 revealed by proxy records. *Nature Geoscience*:1–7.

12. Zielinski GA (1995) Stratospheric loading and optical depth estimates of explosive volcanism over the last 2100 years derived from the Greenland Ice Sheet Project 2 ice core. *Journal of Geophysical Research* 100(D10):20937–20955.

13. Sohn RA, Menke W (2002) Application of maximum likelihood and bootstrap methods to nonlinear curve-fit problems in geochemistry. *Geochem-Geophys-Geosyst* 3(7):1–17.

14. Toohey M, Sigl M (2017) Volcanic stratospheric sulfur injections and aerosol optical depth from 500 BCE to 1900 CE. *Earth System Science Data* 9(2):809–831.
